# The Pulp and Its Pathology

**Published:** 1907-09-15

**Authors:** Russell W. Bunting

**Affiliations:** Ann Arbor, Mich.


### THE PULP AND ITS PATHOLOGY.

BY RUSSELL W. BUNTING, D. D. S., ANN ARBOR, MICH.

Read before the Southwestern Michigan Dental Society, at Battle Creek, April 9, 1907.

From the pulp and its disturbances arise a large percentage of our cases of dental pathology and, for this reason, I shall take the liberty to recall something of its developement and structure and mention a few points in its pathology which I should like to hear discussed.

The pulp of the tooth is the remains of the dentinal papillae from which the dentin has been developed. Being in the beginning simply a condensation of the connective tissue beneath the enamel organ, it develops on its periphery the long dentin-forming cells, the odontoblasts. These odontoblasts secrete the calcific material which becomes hardened to form the ground substance of the dentin. As the dentin is formed the odontoblasts recede and leave prolongations of themselves in canals forming the dentinal fibrillae and canal. As this process goes on and the tooth takes definite shape the dentinal papilla below begins to be circumscribed and is finally surrounded by the dentin, the only connection with the connective tissue about being through the apical foramen. When the dentin is fully formed the pulp occupies the space remaining in the interior of the tooth, the odontoblasts become quiescent on the periphery of the pulp with their prolongations running completely through the dentin to the enamel.

The bulk of the pulp is one of the few places in the body where the connective tissue has retained its primitive, embryonic form, showing no disposition to form fibrous or other tissue. It is, therefore, soft and gelatinous with little fibrous tissue in youth.

In the dental papillae there is beneath the odontoblasts a rich plexus of capillaries which are supplied by several small arteries. As the pulp is formed the arteries decrease in number until there are but one or two vessels entering the apical foramen which soon after their entrance seem to suffer a decrease in the thickness of their walls. As they grow smaller they almost entirely lose their muscle and the capillaries appear as sinuses lined by a single layer of endothelium. No lymphatics have been demonstrated in the pulp. However, Burchard and others have described sinusoidal spaces lined by endothelium containing but few blood corpuscles, which may have the character of lymphatics. The nerves of the pulp are of two kinds, vasomotor to the walls of the blood vessels and sensory to the periphery of the pulp, where they end in knob-like endings near and between the odontoblasts.

Inasmuch as the pulp is composed of a very low order of connective tissue, containing blood vessels with thin walls and no lymphatics, the reason for the many circulatory and infective disturbances is readily understood. When the pulp is irritated in any way, there follows a hyperemia with an enlargement of the small arteries, veins and capillaries, and if the apical foramen is small, the enlargement of the incoming artery presses upon the outgoing vein preventing the free exit of the blood and producing a passive as well as active hyperemia. This condition results in a high pressure of blood in the vessels of the pulp and, if it continues for some time, there will be diapedesis of the blood constitutents through the thin walls of the vessels to the surrounding tissue of the pulp and the inflamation will be inaugurated. This process may go on to complete stasis of the circulation and death of the pulp, which, if there is no infection, results in the dry gangrene of the pulp which we so often see—a simple drying and shriveling of the pulp. However, in any of these stages there is quite likely to be infection. Infection, Miller says, may take place in one of three ways, namely, through the *circulation*, through the *pericementum* and through the *dentin*. We have all seen cases of perfectly sound teeth which, upon being opened, gave forth a vile odor and every indication of putrid gangrene. The source of the organisms could not well be explained except that they came through the blood system. However, the source of infection through the dentin is by far the most common. Hiller says that germs may enter the pulp from a cavity when there is from $\frac{1}{4}$ to $\frac{1}{8}$ mn. of dentin completely covering the pulp. Probably a large number of the mouth germs and germs of decay are capable, upon entrance to the pulp chamber, of producing suppuration of the pulp.

This suppuration may be of two kinds, *ulceration* and *abscess*. Of the two the former is by far the more common. The *ulceration* usually starts upon the surface below the point of inoculation and progressively destroys and liquefies the pulp. The second and less common form, that of *abscess*, may be seen in definite circumscibed infection usually in one horn of the pulp, which soon spreads by metastasis to form secondary abscesses in various parts of the pulp and eventually destroy it. There may be also an infective inflamation of the pulp in which we have no pus formed, only gases, these particular germs not having the property of liquefying gelatin or of forming pus.

We have then in every case of pulp pathology a variety of possibilities to consider. When the pulp is alive and has a history of pain, we must carefully diagnose the condition whether it be simply an acute hyperemia or whether it has gone on to inflamation or chronic hyperemia. When we test with stimulants and get quick, sharp responses which soon subside we think that the pulp is not in as bad condition as when the response is delayed and of a lancinating, throbbing character, continuing for some time after the application. However, we may often be deceived in our symptomatology by the presence of a secondary deposit of the dentin which the pulp has thrown up to protect itself, or by the presence of calcific bodies in the pulp.

When the pulp is exposed we should carefully consider whether the infection is superficial and may possibly be reached by antiseptics, whether there is an abscess present that may possibly be excised, or whether the infection is so deep seated that the whole pulp should be extirpated. In this great care should be exercised, for all of us who attempt to save pulps make mistakes, and those of us who do not value the life of a pulp probably make an equal number of blunders. The pulp with its defective circulation and absence of lymphatics makes an admirable breeding ground for germs when once they have been planted, so that it behooves us to see that the infection is not too deep to be beyond the reach of our antiseptics if we attempt to cap an infected pulp.

When the pulp is completely dead and we attempt to remove it we have a task that we should approach with caution. There is in a gangrenous pulp enough infective material to infect the whole body. It is, moreover, enclosed in a bony tube with an opening at the distal end, directly communicating with the apical tissue. When we open into this infected pulp with a bur we are at the very outset liable to produce the action of a piston and force the infective material through the apex. Also, in every step of the process, drills, broaches, reamers or what not, are all likely to produce this piston-like action. It therefore seems more feasible to avoid instrumentation at the beginning as much as possible and resort to gentle washing of the material by antiseptic solutions and, if necessary, the sealing of strong antiseptics in the pulp chamber to thoroughly sterilize the contents of the canals before attempting their removal.

Gangrenous pulps lying unremoved or their faulty removal frequently give rise to an infection of the apical tissue and furnish the inception of an apical abscess. The germs present in the pulp or their ptomains probably wander out through the apical foramin and find in the apical tissue a favorable lodgement or place of least resistance. These acting upon the tissue cause its death and desolution.- Just why these germs and ptomains should always lodge in the pericemental tissue and not find their way to regions beyond and there elaborate their process is not very clear. We know that the circulation to the pericenental membrane is very rich, there being three distinct sets of arteries supplying it; namely, small arteries given off from the vessels entering the apical foramin to the pulp, small arteries from the alveolus about, and third, those which come down from the periosteum over the alveolus. So that if the circulation were disturbed in the vessels which came from the apical artery, a good collateral circulation should be readily established which should be able to carry off a certain amount of the germs and poisons when they escape from the apical foramen. However, we have all seen many cases of apical inflamation which had the direct history of the presence of a putrid gangrenous pulp for some time; and the apical infection from it appearing simultaneously with the patient's "taking a cold" or other illness which has lowered the body vitality. So that we may infer that the apical tissue normally has a certain amount of resistance and can conduct away a certain amount of noxious substance; but, if the amount is too great or its own vitality is impaired through general or local causes, it may offer assistance where the infection from the pulp may gain foothold and elaborate its toxins.

